# TDP-43 Inhibits NF-κB Activity by Blocking p65 Nuclear Translocation

**DOI:** 10.1371/journal.pone.0142296

**Published:** 2015-11-16

**Authors:** Jingyan Zhu, Max S. Cynader, William Jia

**Affiliations:** 1 Brain Research Center, University of British Columbia, 2211 Wesbrook Mall, Vancouver, BC V6T2B5, Canada; 2 Department of Surgery, University of British Columbia, Vancouver, BC, Canada; Universidade de São Paulo, BRAZIL

## Abstract

TDP-43 (TAR DNA binding protein 43) is a heterogeneous nuclear ribonucleoprotein (hnRNP) that has been found to play an important role in neurodegenerative diseases. TDP-43’s involvement in nuclear factor-kappaB pathways has been reported in both neurons and microglial cells. The NF-κB pathway targets hundreds of genes, many of which are involved in inflammation, immunity and cancer. p50/p65 (p50/RelA) heterodimers, as the major Rel complex in the NF-κB family, are induced by diverse external physiological stimuli and modulate transcriptional activity in almost all cell types. Both p65 and TDP-43 translocation occur through the classic nuclear transportation system. In this study, we report that TDP-43 overexpression prevents TNF-α induced p65 nuclear translocation in a dose dependent manner, and that this further inhibits p65 transactivation activity. The inhibition by TDP-43 does not occur through preventing IκB degradation but probably by competing for the nuclear transporter-importin α3 (KPNA4). This competition is dependent on the presence of the nuclear localization signal (NLS) in TDP-43. Silencing TDP-43 using a specific siRNA also increased p65 nuclear localization upon TNF-α stimulation, suggesting that endogenous TDP-43 may be a default suppressor of the NF-κB pathway. Our results indicate that TDP-43 may play an important role in regulating the levels of NF-κB activity by controlling the nuclear translocation of p65.

## Introduction

TAR DNA binding protein 43 (TDP-43) has recently been implicated in multiple neurodegenerative diseases. It was initially identified as a major protein constituent of the intracellular ubiquitin-positive insoluble neuronal inclusions in both Frontotemporal lobar degeneration (FTLD) and Amyotrophic lateral sclerosis (ALS) [1,2]. Since then, many other neurodegenerative disorders were reported with TDP-43 dysfunction as one of clinic pathological features, including Alzheimer’s Disease (AD) [3]; Parkinson’s disease (PD) [4] and Huntington’s disease [5]. In a recent case study, FTLD with abnormal cytoplasmic localization of TDP-43 (FTLD-TDP) even showed systemic inflammation and elevated plasma TNF-α concentration [6].

TDP-43 is a RNA-binding nuclear protein that is abundantly expressed in almost all mammalian tissues [7]. It is a 414-amno acid protein of 43 kDa encoded by six exons of the TARDBP gene. It consists of two RNA recognition motifs (RRM1 and RRM2) and a carboxy-terminal glycine-rich domain that are characteristic of ribonucleoproteins (hnRNPs) [8]. Many clinical mutations have been identified to remarkably concentrate in the C-terminal glycine-rich domain, which has been reported to mediate protein-protein interaction and interact with hnRNP family members in RNA splicing inhibitory activity [9].

The N-terminal region of TDP-43 contains a nuclear localization signal (NLS) and a nuclear export signal (NES) that regulate the translocation of TDP-43 between the cytoplasm and the nucleus [10]. Nuclear translocation of proteins containing a NLS occurs by means of specific carriers that are termed as karyopherins, including importins and exportins for nuclear import and export, respectively [11]. Importin α is an adaptor protein that recognizes the nuclear localization signal (NLS) within the cargo proteins and its N-terminal importin β-binding domain (IBB) directly dimerizes with importin β, which interacts with nuclear pore complexes (NPC) in the nuclear envelope to allow entering into the nucleus [11]. The presence of an NLS suggests that TDP-43 may interact with importins for its nuclear translocation. Indeed, knockdown of importin β results in redistribution of TDP-43 to the cytoplasm [12]. Furthermore, TDP-43 has been shown to bind with importin α1, importin α3, importin α4, importin α5, importin α7 and importin β by in vitro GST pull down assays [13], indicating that TDP-43 nuclear transportation might be through the classic nuclear import pathway by binding to importin α isoforms.

Another well-known protein that relies on the classic nuclear import pathway for its nuclear localization is NF-κB. NF-κB is an important transcriptional factor regulating expression of many genes. Upon activation by various cell surface factors, NF-κB translocates to the nucleus, which can be measured by the nuclear presence of its subunit p65. The p65 subunit of NF-κB possesses an NLS as well. It has been shown that p65 nuclear transportation occurs through binding with importin α3 (KPNA4) when NF-κB is released from IκB [14]. Thus, the importin transportation system plays an important role in NF-κB signaling by mediating its transport to the nucleus. The NF-κB pathway targets more than hundreds of genes, of which many are involved in inflammation, immunity and cancer. Moreover, experiments have shown that inhibition of NF-κB leads to loss of antiapoptotic protein expression, such as Bcl-2, cellular-inhibitor of apoptosis 2 (c-IAP-2) and Bcl-xL [15]. Due to the critical role of NF-κB in regulation of cell death, angiogenesis and resistance to anticancer therapies, numerous inhibitors targeting the NF-κB pathway are under development or already in the clinic [16,17].

In the meanwhile, there is evidence showing that different proteins can be recognized specifically by only one importin α or one importin subset [18,19]. Importantly, it has been demonstrated that importin α could be saturated by peptides containing NLS and the binding abilities to importin α were competitive [20]. Given the fact that TDP-43 and NF-κB share the same classic nuclear transportation mechanism, we hypothesized that the two proteins may functionally interact to each other by competing for access to the nuclear transportation machinery. TDP-43 has been shown to regulate NF-κB signal pathway in neurodegenerated diseases [21]. Since TDP-43 is a widely distributed RNA binding protein in many tissues and given the central role of NF-κB in many cellular functions, we were asking if TDP-43 has the same effect in non-tumoural cells or not. Here we show that overexpression of TDP-43 reduced NF-κB activity in human breast cancer MCF-7 cells and we further report that TDP-43 prevents the nuclear transportation of p65 by competing for importin alpha 3 (KPNA4).

## Results

### 1. TDP-43 overexpression blocked p65 nuclear translocation in a dose dependent manner

**Fig 1:**
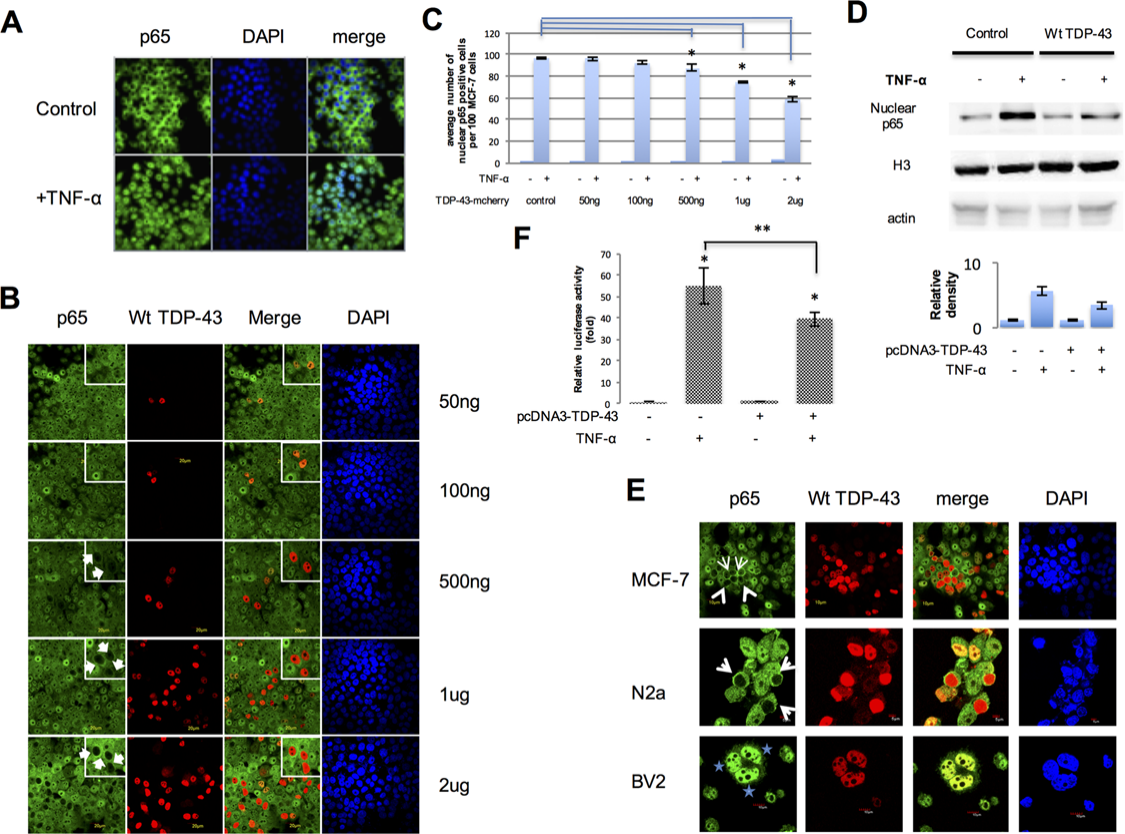
Overexpression of TDP-43 on p65 activation. **A**. p65 (Green) is translocated into the cells’ nuclei (Blue) after 30 minutes of 10ng/ml TNF-α treatment (n = 3 repeats). **B**. MCF-7 cells were transfected with various doses (indicated on the right) of wild type TDP-43 tagged with mCherry expressing plasmids (middle panel, red) and were treated with 10ng/ml TNF-α for 30 minutes. In the top right corner, insets of higher magnification show the localization of p65 and nuclear TDP-43 staining (n≥3 independent experiments). **C**. The quantification of B. **D.** MCF-7 Cells were transfected with 4μg plasmid encoding TDP-43 tagged with mCherry or mCherry empty plasmid (control) and treated with 10ng/ml TNF-α for 30 minutes. Nuclear proteins were isolated and detected by western immunoblotting. Histone H3 was used as a nuclear marker. The relative density was the average of 3 individual experiments. **E**. MCF-7, Neuro 2a and BV2 cells were transfected with Wild type TDP-43 tagged with mCherry expressing plasmids (middle panel, red). MCF-7 and Neuro 2a cells were treated with 10ng/ml TNF-α for 30 minutes; BV2 cells were treated with 10μg/ml LPS for 30 minutes. Arrows indicate the blocked p65 nuclear translocation by Wt TDP-43 and stars indicate the normal p65 nuclear translocation with Wt TDP-43 overexpression. n≥3 independent experiments. **F**. The plasmid encoding TDP-43 tagged with mCherry or mCherry empty plasmid (control) was cotransfected along with NF-κB-luc (containing the wild type NF-κB-binding site). Cells were treated with 10ng/ml TNF-α for 30 minutes. Luciferase activity was measured after 24 hours. The plotted error bars represent mean±SEM from 3 independent experiments; *p < 0.05 compared to control, one-way ANOVA. ** p < 0.05 compared to the TNF-α treated control samples, Student's t test.

MCF-7 cells were treated with 10ng/ml TNF-α for 30 minutes followed by immunofluorescence using an antibody for p65. Upon treatment with TNF-α, p65 was found to be translocated from the cytoplasm to the nucleus (Fig 1A). We then transfected the cells with different amounts of wild type TDP-43 tagged with mCherry expressing plasmids (50ng, 100ng, 500ng, 1μg and 2μg) followed by treatment with the same concentration of TNF-α. As shown in Fig 1B, the nuclear localization of p65 was still seen in most of cells transfected with 50ng and 100ng TDP-43 DNA. The p65 and TDP-43 were co-localized in the nucleus in a few cells. However, the translocation of p65 was significantly blocked in cells transfected with higher concentrations of the TDP-43 expressing plasmid (500ng, 1μg and 2μg) (Fig 1C). Our results show that p65 nuclear transport is blocked in cells overexpressing TDP-43 (Arrows in Fig 1B).

To quantitatively confirm that p65 nuclear translocation was inhibited by overexpressing TDP-43, nuclear proteins were extracted and p65 protein levels were quantified by western blotting (Fig 1D). After TNF-α treatment, nuclear p65 protein level was dramatically increased compared to untreated cells. In the cells transfected with 4μg wild-type TDP-43 expressing plasmids, the TNF-α induced increase of nuclear p65 protein was significantly less than the controls. Histone H3 was used as a marker for nuclear proteins.

### 2. The blockade of p65 nuclear translocation by TDP-43 overexpression is cell type specific

Next, we tested whether the phenomenon that TDP-43 overexpression blocked p65 nuclear translocation is cell type specific. MCF-7, Neuro 2a and BV2 cells were transfected with Wild type TDP-43 tagged with mCherry expressing plasmids. p65 nuclear translocation in MCF-7, Neuro 2a and BV2 cells was stimulated with either TNF-α or LPS. The results showed that the blockade of p65 nuclear translocation by TDP-43 overexpression happened in MCF-7 and Neuro 2a cells (Arrows in Fig 1E) but not in BV2 cells (Stars in Fig 1E). These results suggest that this phenomenon might be cell-type specific.

### 3. Overexpression of TDP-43 inhibits NF-κB transactivation activity after TNF-α treatment

To further confirm that inhibition in p65 nuclear translocation by TDP-43 overexpression results in reduced activity of NF-κB, we used a construct containing a luciferase reporter gene driven by an NF-κB response element (κB site) [22]. The results showed that luciferase activity in cells co-transfected with both the reporter gene construct and TDP-43 was significantly lower than that of controls (-15.33+/-5.38 fold), indicating that overexpression TDP-43 in those cells indeed suppressed TNF-α induced transactivation activity of NF-κB (Fig 1F), which was consistent with the results of the p65 nuclear translocation experiments. The data were expressed as mean ± standard error mean (S.E.M) from three independent experiments.

### 4. Knockdown of endogenous TDP-43 increases the nuclear p65 protein level after TNF-α treatment

**Fig 2:**
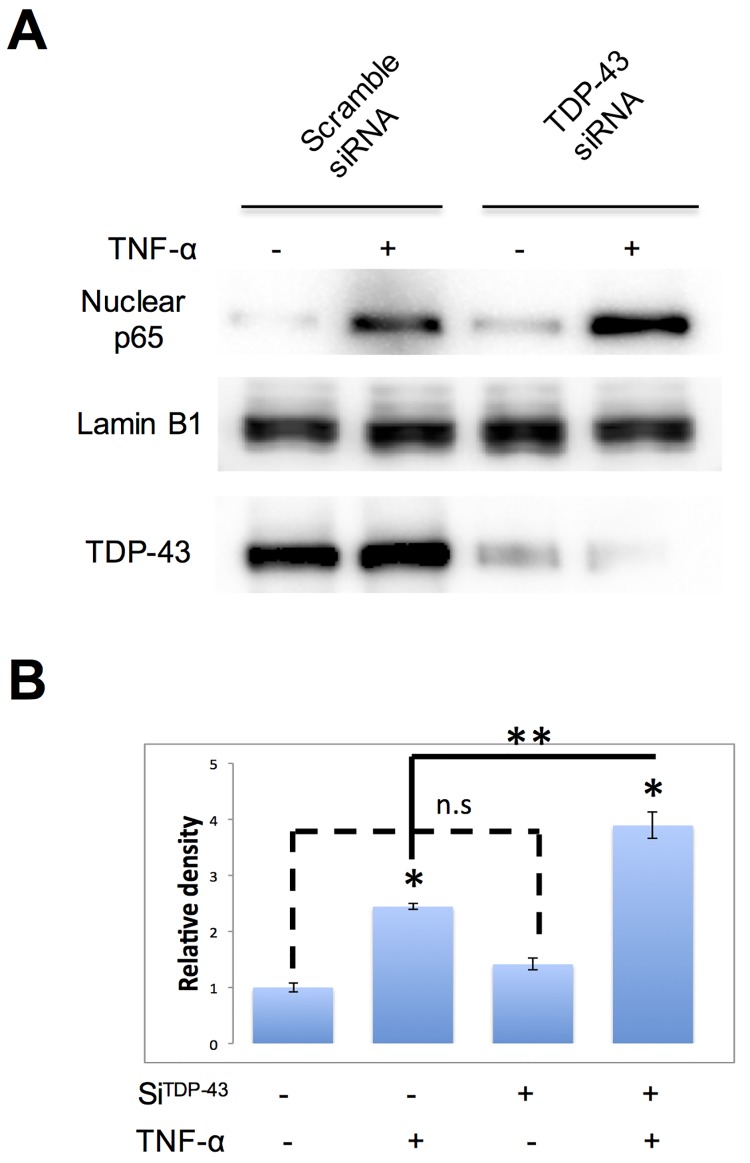
Knockdown of endogenous TDP-43 increases nuclear p65 protein levels after TNF-α treatment. **A**. MCF-7 cells were transfected with TDP-43 siRNA or scrambled siRNA, treated with 10ng/ml TNF-α for 30 minutes. Nuclear proteins were analyzed by western blot and detected with antibodies against TDP-43, p65 and Lumin B1 as indicated. Lumin B1 was used as nuclear marker. n = 3 independent experiments. **B.** The relative density of p65 as shown in A. n.s not significant, *p < 0.05 compared to control, one-way ANOVA, ** p < 0.05.

To ask whether the inhibition of NF-κB activity by TDP-43 overexpression is an artifact of high dose of exogenous TDP-43 or whether it is a normal function of TDP-43, we used a short interference RNA (siRNA) to suppress endogenous TDP-43 expression by targeting the 3’UTR region of human TDP-43 mRNA. Control cells were transfected with a scrambled siRNA. The TDP-43 protein level of the knockdown group was dramatically decreased (63.52+/-5.76%) comparing to the control group. Knockdown of TDP-43 did not affect the subcellular distribution of p65 under resting conditions, but significantly enhanced nuclear p65 translocation when the cells were stimulated with TNF-α (Fig 2A and 2B), suggesting that endogenous TDP-43 may suppress stimulated p65 nuclear transportation.

### 5. Overexpression of TDP-43 accelerates IκB degradation

**Fig 3:**
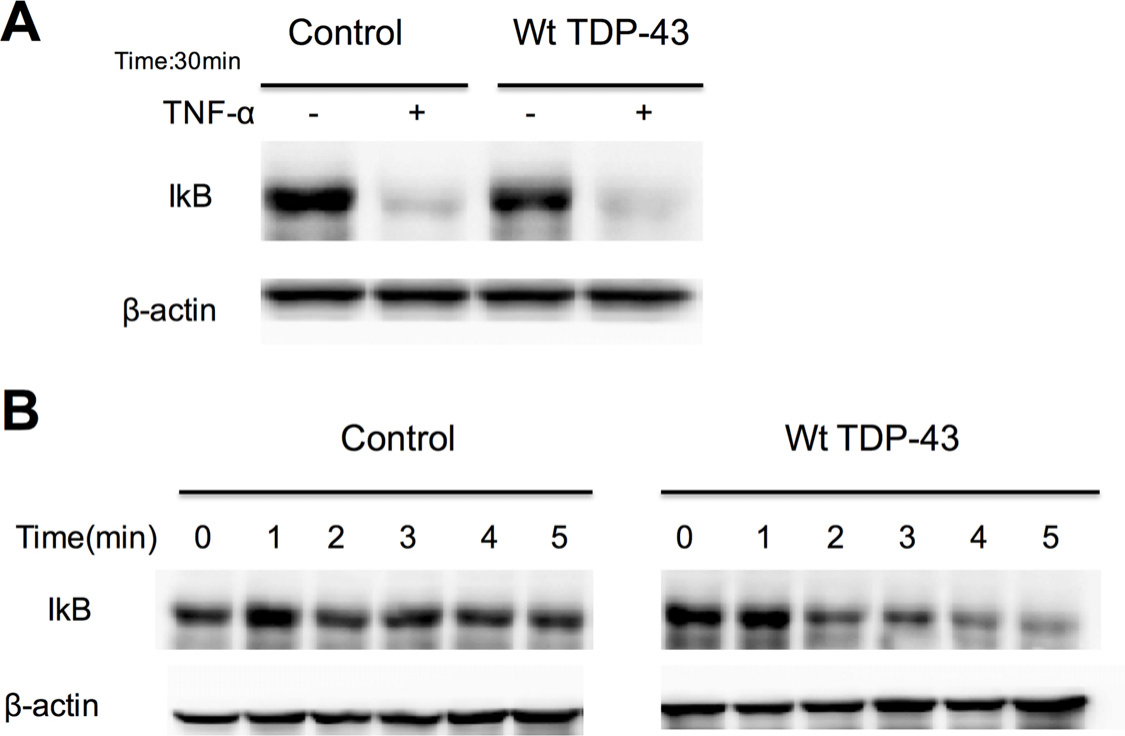
Overexpression of TDP-43 accelerates IκB degradation. Cells were transfected with wild type TDP-43 expressing plasmids (tagged with mCherry) or mCherry empty plasmid (control) and were treated with 10ng/ml TNF-α for the indicated periods. Total proteins were extracted for Western blot analysis using antibodies as indicated. n = 3 independent cultures; **A**. 30 min after TNF-α treatment, the degradation of IκB occurred in both control group and TDP-43 overexpression group. **B**. Overexpression of TDP-43 facilitates the degradation of IκB within 5 minutes after TNF-α treatment.

Stimulated p65 nuclear translocation requires that the protein be released from the p65/IκB complex. Upon stimulation with TNF-α or LPS, IκB is phosphorylated and disassociates from p65, followed by degradation [23]. To ask whether this is part of the mechanism by which TDP-43 overexpression causes NF-κB inhibition, we first measured IκB levels. Overexpression of TDP-43 had no effect on IκB level in cells without TNF-α treatment (Fig 3A). Surprisingly, overexpression of TDP-43 increased the rate of IκB degradation upon stimulation by TNF-α (Fig 3B). Therefore, reduced nuclear translocation of p65 by overexpressing TDP-43 is not due to inhibition of IκB degradation to maintain the p65/IκB complex in the cytoplasm.

### 6. Overexpressing TDP-43 inhibits NF-κB activity by competing for the nuclear transporter system

**Fig 4:**
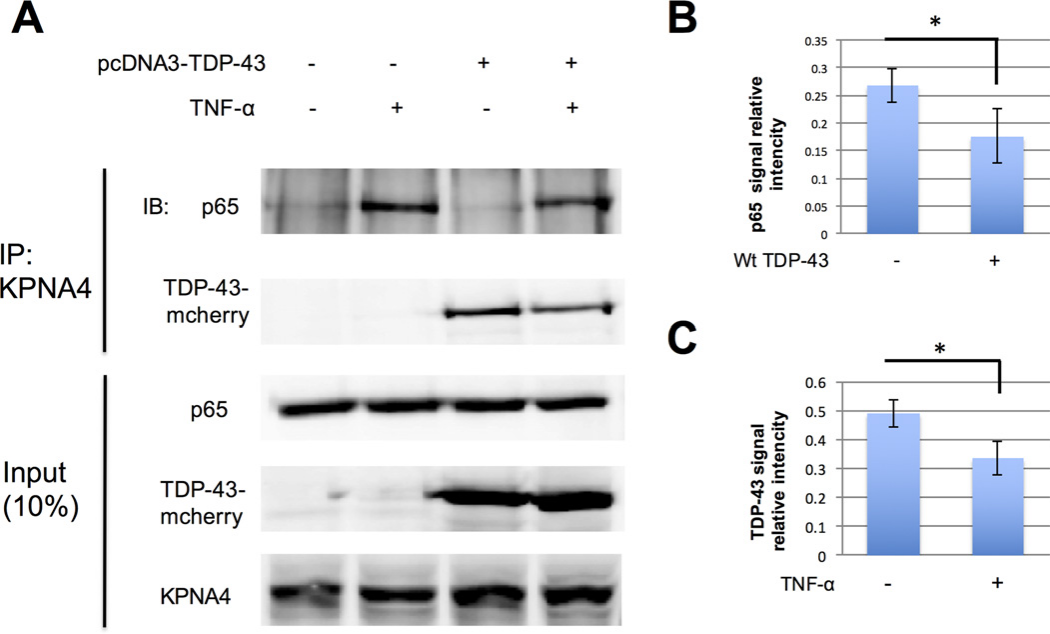
Overexpressing TDP-43 inhibits NF-κB activity by competing for the nuclear transporter. **A**. Cells were transfected with wild type TDP-43 expressing plasmids (tagged with mCherry) or mCherry empty plasmid (control) and were then treated with 10ng/ml TNF-α for 30 minutes. Cell lysates were subjected to coimmunoprecipitation and western blot for the presence of p65 and TDP-43. 10% cell lysate were reserved as input. p65, TDP-43 and importin α3 (KPNA4) were used to detect input. n = 3 repeats. **B**. The relative density of p65 as shown in A (IP), *p<0.05. **C**. The relative density of wild type TDP-43 as shown in A (IP), *p<0.05.

Previous studies have shown that NF-κB is transported into the nucleus by importin α3 (KPNA4) [14]. Normally NF-κB located in the cytoplasm is in an inactive form and the NLS of p65 is masked by IκB. Upon stimulation, IκB is degraded and the NLS in p65 is exposed allowing its binding to importin α3 (KPNA4) and its nuclear translocation. To test whether TDP-43 could affect the interaction between p65 and importin α3 (KPNA4), we used co-immunoprecipitation to measure the association of p65 and importin α3 (KPNA4). As shown in Fig 4, without the stimulation, TDP-43 but not p65 was strongly associated with importin α3 (KPNA4). Upon TNF-α treatment, p65 drastically increased its association with importin α3 (KPNA4). However, when TDP-43 was overexpressed in the cells, the amount of p65 associated with importin α3 (KPNA4) was significantly reduced and was about the same as TDP-43. Thus, these results suggest that TDP-43 may compete with p65 for the binding to importin α3 (KPNA4) nuclear transporter to reduce the translocation and hence the nuclear presence of p65.

### 7. The blockade of NF-κB nuclear translocation by TDP-43 can be prevented by overexpression of p65

**Fig 5:**
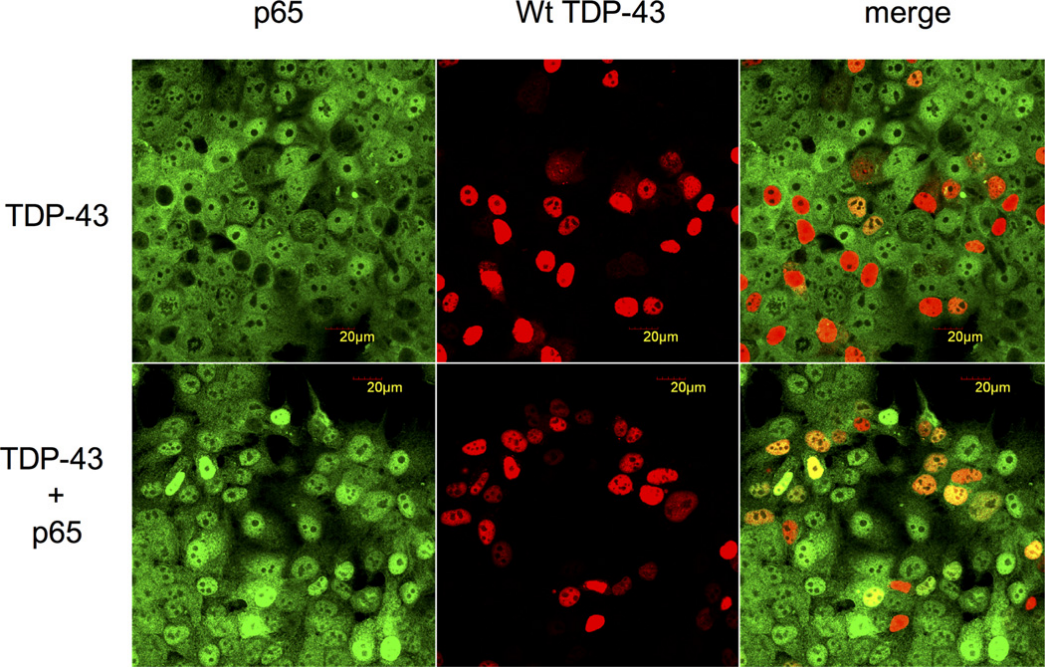
The blockade of NF-κB nuclear translocation by TDP-43 can be prevented by overexpression of p65. MCF-7 cells were transfected with wild type TDP-43 expressing plasmids (top row) alone or cotransfected with TDP-43 and p65 expressing plasmids (bottom row). Then MCF-7 cells were treated with TNF-α for 30 minutes. The p65 was found co-localized with TDP-43 in the nuclei (bottom row). n = 3 independent experiments.

To verify whether the inhibition by TDP-43 for p65 binding to importin α3 (KPNA4) nuclear transporter is indeed a competitive interaction, we co-transfected cells with plasmids expressing both TDP-43 and p65. Cells were transfected with 1μg TDP-43 expressing plasmids and an empty plasmid or with 1μg TDP-43 and 1μg p65 expressing plasmids. 24 hours after transfection, cells were treated with 10ng/ml TNF-α for 30 minutes, then fixed and probed with p65 antibody. As shown in Fig 5, p65 was strongly present in the nucleus in most p65 and TDP-43 co-transfected cells.

### 8. The NLS domain in TDP-43 is critical for its competition with p65 nuclear translocation

**Fig 6:**
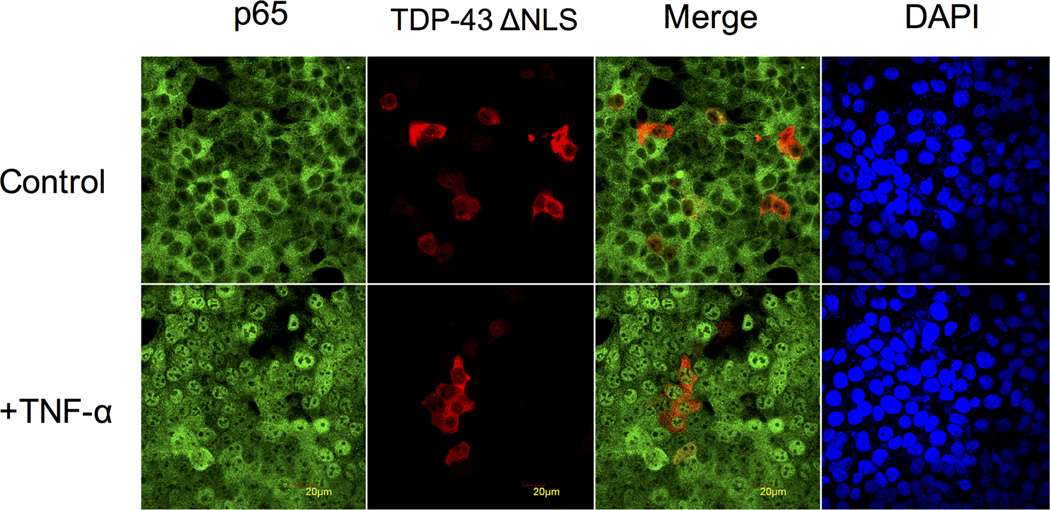
The NLS mutated TDP-43 does not affect p65 nuclear translocation. MCF-7 cells were transfected with TDP-43 ΔNLS expressing plasmids (middle panel, Red). After 30 minutes of TNF-α treatment, labeled p65 (Left panel, Green) was found in the nuclei. n = 3 independent experiments.

To confirm whether the NLS of TDP-43 was responsible for the competition between TDP-43 and p65 for the nuclear transportation system. We mutated three amino acids in the TDP-43 NLS sequence, which abolishes the ability of TDP-43 to bind to KPNAs [10, 24]. We transfected the NLS mutated TDP-43-expressing plasmids followed by stimulation with TNF-α (Fig 6). As expected, the NLS mutated TDP-43 stayed in the cytoplasm. More importantly, nuclear translocation of p65 upon stimulation was not affected by overexpression of the mutant TDP-43 at all. These results clearly demonstrated that the NLS sequence of TDP-43 is critical for its ability to compete with p65 for nuclear transportation.

### 9. The competition of importin α3 (KPNA4) happens in both cytoplasm and nucleus

**Fig 7:**
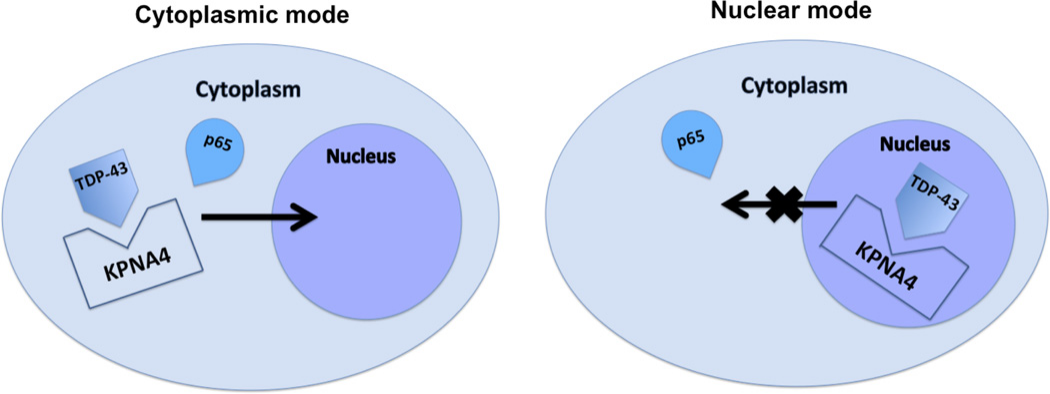
Schematic hypothetical modes for TDP-43 and p65 competition. The presence of TDP-43 in the cytosol may compete with p65 for importin α3 (cytoplasmic mode) and therefore to inhibit the nuclear localization of p65. An alternative mode (nuclear mode) shows that nuclear TDP-43 may occupy importin α3 to prevent its exit and therefore to reduce the availability of free importin α3 in the cytosol for p65 nuclear translocation.

**Fig 8:**
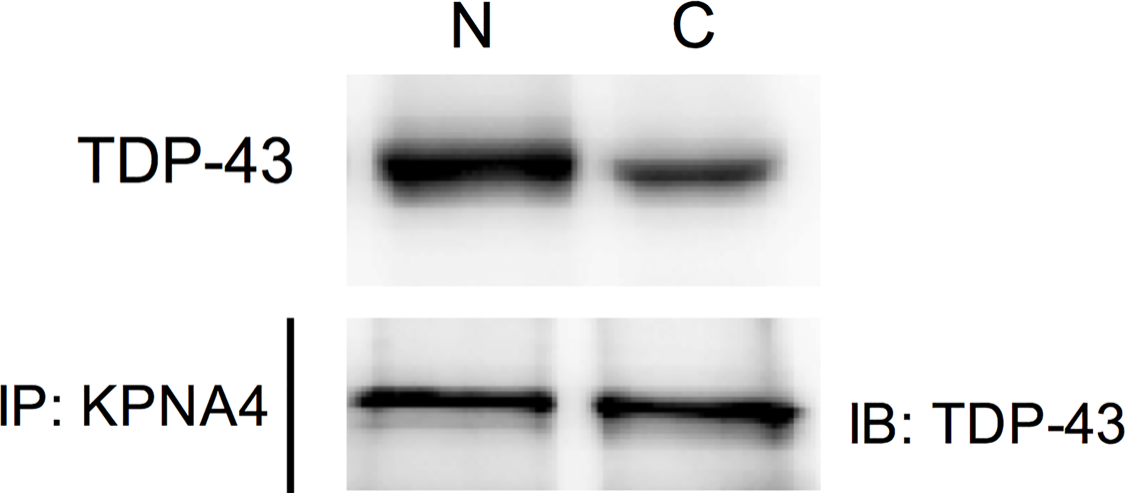
The competition of importin α3 (KPNA4) occurs in both the cytoplasm and nucleus. The nuclear and cytoplasmic proteins of MCF-7 cells were separated after overexpression of wild type TDP-43 and TNF-α stimulation. The top panel shows the protein levels of total TDP-43 in each fraction. N: nucleus. C: cytoplasm. The bottom shows that TDP-43 interacted with importin α3 (KPNA4). p < 0.05, Student's t test.

There are two hypothetical modes to explain how TDP-43 can compete with p65 for nuclear translocation (Fig 7). The first possible mode (cytoplasmic mode) is that TDP-43 competes with p65 for importin α3 (KPNA4) in the cytoplasm, which reduces the efficiency of p65 nuclear transportation. The second mode (nuclear mode) is that TDP-43 binds to nuclear importin α3 (KPNA4) and retains the transporter in the nucleus, which reduces the concentration of importin α3 (KPNA4) in the cytoplasm resulting in less p65 nuclear translocation. To answer this question, we immunoprecipitated importin α3 (KPNA4) in both nuclear and cytoplasmic cellular fractions followed by blotting for TDP-43 in cells stimulated with TNF-a. As shown in Fig 8, even though TDP-43 was predominantly located in the nucleus (Fig 8 top), the interaction between TDP-43 and importin α3 (KPNA4) occurs both in the nucleus and cytoplasm (Fig 8 bottom). Thus, both modes proposed above may co-exist.

## Discussion

In this study, we used multiple approaches to demonstrate that TDP-43 regulates p65 nuclear translocation through binding to the transporter importin α3 (KPNA4), thus inhibiting the activity of the canonical NF-κB pathway. We first showed that p65 failed to translocate into the nucleus in TDP-43 overexpressing cells in a dose dependent manner using immunocytochemistry and western blots. To rule out the possibility that the mCherry tag might affect p65 translocation, we transfected an empty plasmid pcDNA3-mCherry as a control (S1 Fig). The mCherry empty plasmid did not show any effects on p65 intracellular localization with or without TNF-α treatment.

Functionally, the reduced nuclear localization of p65 mediated by TDP-43 competition was associated with reduced activity of transactivation go accessed with NF-κB reporter assays. We have also concluded that the blockade of p65 nuclear translocation by overexpressing TDP-43 is attributable to the binding of TDP-43 to nuclear transporter importin α3 (KPNA4) through its NLS. This is supported by the following evidence: 1) Overexpression of TDP-43 does not inhibit but rather accelerate the degradation of IκB; 2) Association of p65 with its nuclear transporter importin α3 (KPNA4) was reduced in the presence of overexpressed TDP-43 using a co-immunoprecipitation assay; 3) the inhibition of p65 nuclear translocation by overexpressed TDP-43 can be completely reversed by simultaneous overexpression of p65; 4) A mutant TDP-43 that lacks NLS was completely unable to inhibit p65 nuclear translocation.

It has been reported that TDP-43 and p65 may bind to each other in the nucleus [21]. Therefore, a possible explanation for lack of nuclear p65 in TDP-43 overexpressing cells is because that the antibody we used could not recognize the p65 in the nuclei as the antibody epitopes in p65 might be masked by overexpressed nuclear TDP-43. However, this is an unlikely possibility since western blots also showed reduced nuclear p65 (Fig 1D). In addition, the luciferase activity (Fig 1F) of the transactivation assay was also reduced. Finally, when we silenced the endogenous TDP-43, the nuclear p65 protein level was increased (Fig 2A and 2B).

It is interesting that overexpression of TDP-43 caused accelerated IκB degradation shortly after TNF-α stimulation but not in the unstimulated cells. It is not known how TDP-43 does that nor whether this is an artifact due to overexpressed TDP-43. The latter can be verified by comparing the rate of IκB degradation upon TNF-α stimulation in cells with or without reduced endogenous TDP-43 by siRNA knockdown. Interestingly, overexpression of TDP-43 does not change the level of IκB in unstimulated cells. This suggests that overexpressed TDP-43 may only selectively accelerate the degradation of phosphorylated IκB.

Since both subunits of p50 and p65 contain the NLS sequence, and only the NLS in p65 is masked by IκBα [14], it has been proposed that the p50/p65/IκBα complex is continuously shuttled between the nucleus and cytoplasm under unstimulated conditions [25]. Since it is not clear whether nuclear translocation of the inactivated p50/p65/IκBα complex is also dependent on importin α3 (KPNA4), the effect of overexpressed TDP-43 on transportation of the NF-κB complex in the resting status remains to be confirmed although it is apparent that total p65 in the nuclei was not significantly higher in TDP-43 k/o cells without TNF-α stimulation (Fig 2A), suggesting that TDP-43 may not significantly interfere with the nuclear transportation of the inactivated p50/p65/IκBα complex.

It is worth pointing out that there is a large amount of TDP-43 associated with importin α3 (KPNA4) in unstimulated cells (Fig 4A). Unlike overexpression of TDP-43 for p65, overexpression of p65 did not change the nuclear localization of endogenous TDP-43 even though more nuclear localized p65 was observed. Therefore, it seems that the binding to importin α3 (KPNA4) by TDP-43 may only affect the activity of NF-κB but not vice versa. Since GST pull down experiments have revealed that TDP-43 can bind to importin α1, 3, 4, 5, 7 and importin β [13], an obvious explanation is that TDP-43 is transported by multiple nuclear transporters while importin α3 (KPNA4) is the major one for NF-κB in the MCF-7 cells used in the experiments. TNF-α treatment did not affect TDP-43 intracellular distribution (S2 Fig). The cell-type specific response to TDP-43 mediated blockade on p65 nuclear translocation found in this study might coincide with a previous study [21,26] showing that TDP-43 co-localized with p65 in the nucleus in BV2 cells. However, since we observed in previous experiments that low dose Wt TDP-43 did not affect p65 nuclear translocation, we cannot exclude the possibility that the lack of response to TDP-43 in BV2 cells was due to low concentration of TDP-43 as BV2 cells are difficult to be transfected. In addition, it is also possible that differences in the stimulus could also affect the mechanisms of p65 nuclear translocation, which may attribute to lack of effect of TDP-43 in BV cells.

It is important to note that p65 level was increased in the nuclei upon TNF-α stimulation when we knocked down endogenous TDP-43 using a siRNA. This suggests that p65 nuclear translocation is constantly inhibited by TDP-43 and therefore, that TDP-43 may act as a default negative regulator on NF-κB transactivation activity. Thus, up-regulation of TDP-43 could be a therapeutic strategy for a wide range of human cancers in which NF-κB is constitutively activated [27,28] and is related to various oncogenic phenotypes, such as angiogenesis, tumor cell survival, cancer invasion and inflammation in tumor microenvironment [29]. Our finding that TDP-43 constitutively inhibits NF-κB pathway by blocking nuclear transportation of p65 suggests a novel mechanism of a potential anti-cancer and anti-inflammation treatment by elevating TDP-43.

## Materials and Methods

### Cell culture and transfection

Human breast cancer (MCF-7) cells, mouse neuroblastoma cells (Neuro-2a) and mouse microglia cells were obtained from the American Type Culture Collection. The cells were maintained in Dulbecco's modified Eagle's medium (DMEM; Sigma Chemical Co., St. Louis, MO) supplemented with 10% fetal bovine serum (FBS) (GIBCO BRL, Grand Island, NY) and 1% antibiotics (Gibco-BRL, Grand Island, NY). Cultures were maintained at 37°C in a humidified incubator (NuAir, Plymouth, MN) with 5% CO2 and were passaged every 3–4 days. The plasmids were transfected into the MCF-7 cells using the Polyethylenimine (PEI) transfection system. Briefly, using 6 well plates, 4μg DNA and 16μl PEI were diluted separately in 250μl opti-MEM Reduced Serum Medium (Invitrogen, Carlsbad, CA) per well. After 5 minutes of incubation, the diluted DNA and PEI were combined, mixed gently and allowed to stand for 15 minutes at room temperature. Thereafter, 500μl Opti-MEM І Reduced Serum Medium was added to the DNA-PEI mixture and mixed well. Then, a total of 1ml of the mixture was added into each well of the 6-well dishes with the cultured cells. After 5 hours of incubation, 1ml of fresh DMEM containing 10% fetal bovine serum was added into the wells. The cells were returned to the incubator for an additional 24 hours.

### Plasmid construction and siRNA preparation

TDP-43 ΔNLS plasmid: The site- directed mutations was generated using wild type TDP-43 plasmid as template. Three amino acids were changed: K82A, R83A, K84A, which are the first three amino acids of the TDP-43 NLS sequence that have been shown to be essential for cytoplasm in transport [10]. The PCR product was subcloned into a mCherry-pcDNA3 vector using restriction sites HindIII and BamH1. Primers sequences are as follows: Forward GCAGCAGCAATGGATGAGACAGATGCTTCATCA Reverse TGCTGCTGCGTTATCTTTTGGATAGTTGACAAC. The siRNA was designed as described previously [30] and targeted the 3’UTR of human TDP-43 mRNA (GenBank accession no. NM_007375). The sequences are as follows: TDP-43 siRNA sense: 5′-CACUACAAUUGAUAUCAAAUU-3′; antisense: 5′-UUUGAUAUCAAUUGUAGUGUU-3′. The negative control siRNA (scrambled-siRNA) (Life Technologies Co.) was used to account for nonsequence-specific effects. TDP-43 siRNA was suspended in diethyl pyro-carbonate water to a final concentration of 20μM and was transfected using Lipofectamine RNAiMAX reagent (Invitrogen, Carlsbad, CA). The RNA interference effect was detected 48 hours or 72 hours after transfection.

### Tumor necrosis factor-alpha (TNF-α) treatment

Recombinant human TNF-α (R&D systems, Minneapolis, MN) was dissolved in PBS with 0.1% BSA to 100μg/ml. To observe the p65 nuclear translocation, 10ng/ml TNF-α was pre-incubated with the cells for 30 minutes at 37°C. Control cultures (without TNF-α) underwent the same medium changes.

### Immunocytochemistry

The MCF-7 cells were plated and cultured on poly-D-lysine coated glass coverslips at a density of 5 x 10^5^ cells per well in 12-well plates. 24 hours after transfection, the cells were treated with TNF-α for 30 minutes. Then the cells were washed with cold PBS (pH 7.4) 3 times and fixed with 4% Paraformaldehyde (PFA; Sigma, Saint Louis, MO) in PBS (pH 7.4) for 15 minutes at room temperature. They were then rinsed with cold PBS twice and permeabilized with PBS containing 0.25% Triton X-100 (Sigma, Saint Louis, MO) for 10 minutes at room temperature followed by washing with PBS 3 times for 5 minutes. To block the nonspecific binding of the antibodies, samples were incubated with 1% Bovine serum albumin (BSA; Invitrogen, Carlsbad, CA) in PBST (0.1% Tween 20 in 1x PBS) for 30 minutes at room temperature. Primary antibody against p65 (1:1000; Abcam, Cambridge, MA) was added to the samples and incubated for 1 hour at room temperature. After washing with PBS 5 minutes×3 times, the cells were incubated with anti-rabbit secondary antibody conjugated with Alexa 488 (Invitrogen, Carlsbad, CA) for 1 hour at room temperature followed by ×3 five minutes washes with PBS. The coverslips were mounted on glass slides with antifade reagent with DAPI (Invitrogen, Carlsbad, CA). The samples were stored at 4°C in dark. Images were obtained with an Olympus Fluoview FV1000 Confocal scanning microscope.

### Nuclear protein Extraction and BCA protein assay

The nuclear and cytoplasmic proteins were extracted using an NE-PER Nuclear and Cytoplasmic Extraction kit (Pierce, Rockford, IL) according to the manufacturer’s instructions. The whole cell lysate was extracted by 2х sample buffer which containing 62.5mM Tris-HCl (pH 6.8), 2% sodium dodecyl sulfate (SDS), 25% Glycerol, 0.02% (w/v) Bromophenol blue and 5% β-mercaptoethanol. The protein concentrations were determined using a BCA protein assay (Pierce, Rockford, IL) according to the manufacturer’s instructions and were analyzed by measuring the absorbance of each sample at a wavelength of 560nm using a “uQuant” microplate spectrophotometer (Bio-Tek Instruments, USA).

### Western immunoblotting

For each sample, 35ug of protein was separated in 12% SDS-PAGE and transferred to PVDF membranes at 4°C using the Bio-Rad Wet Transfer System (Bio-Rad, Hercules, CA). The membrane was blocked in 5% non-fat milk in TBST (Tris buffered saline tween 20) for 1 hour at room temperature. Then, the membrane was stained with the primary antibody dissolved in 3% BSA-TBST (Sigma, Saint Louis, MO) at 4°C overnight. The primary antibodies used included: Rabbit polyclonal NF-κB p65 antibody (1:2000; Abcam, Cambridge, MA); Rabbit polyclonal Lamin B1 antibody (1:1000; Abcam, Cambridge, MA); Rabbit polyclonal TARDBP antibody (1:1000; ProteinTech, Chicago, IL); Rabbit polyclonal KPNA4 antibody (1:1000; Novus, Littleton, CO); Rabbit monoclonal IκB alpha antibody (1:2000; Abcam, Cambridge, MA); Mouse monoclonal IκB alpha (phospho S32 + S36) antibody (1:500; Abcam, Cambridge, MA); Rabbit polyclonal Histone H3 antibody (1:1000, Cell Signaling, Danvers, MA) and rabbit polyclonal Actin antibody (1:1000, Cell Signaling, Danvers, MA). After washing with TBST, membranes were incubated with HRP-conjugated goat anti- mouse or goat anti- rabbit secondary antibody (1:5000, PerkinElmer Life Sciences) for 1 hour. Densitometric values of targeted proteins were acquired using the enhanced chemiluminescence reaction assay (ECL, PerkinElmer Life Sciences). Calculation of protein density and normalization with actin (or other internal controls) were performed using Image J software (NIH).

### Luciferase assay

An NF-κB-luciferase reporter construct (consensus NF-κB binding sequence) was a generous gift provided by Dr. Song (University of British Columbia, B.C.). Transfections were performed using the Lipofectamine 2000 system (Invitrogen, Carlsbad, CA) according to the manufacturer's protocol. 24 hours after transfection, cells were stimulated with 10ng/ml TNF-α for 30 minutes and were ready for analysis after 24 hours using a luciferase kit (Promega, Madison, WI) used according to the manufacturer’s instructions. The cell lysate was transferred to a pre-chilled microcentrifuge tube followed by brief centrifugation. 20μl cell lysate was mixed with 100μl of luciferase assay reagent and the products were measured in an assay plate (Corning, NY).

### Co-immunoprecipitation

The cells were harvested with gentle lysis buffer (25mM Tris-Hcl, 10mM NaCl, 20mM EDTA, 10mM EGTA, 0.5% Triton-100, 10% Glyceral, 1mM dithiothreitol and protease inhibitor). Then the cell lysates were treated with ultrasound and centrifuged at 4 degrees. The supernatant was pre-cleared using 10μl 50% protein A/G magnetic beads (Themo, Rockford, IL) for 1 hour and incubated with 5μg KPNA4 antibody at 4 degree overnight. 50μl 50% protein A/G magnetic beads were added into the cell lysate for 2 hours on the next day. Then the proteins were eluted from the beads with 35–50μl 2×lysis buffer and boiled for 3 minutes. The samples were further analyzed by western immunoblotting.

### Statistical analysis

All data are expressed as mean ± standard error mean (S.E.M). Student’s t test was used to test statistical significance of the differences in two groups and one-way ANOVA was used to analyze the statistical significance of the differences among three or more groups of data. Statistical significance was considered as p < 0.05. For all experiments, data was obtained from at least 3 replicates of independent cultures.

## Supporting Information

S1 Fig pcDNA3-mCherry empty plasmids does not affect p65 nuclear translocation in MCF-7 cells. MCF-7 cells were transfected with 2μg pcDNA3-mCherry empty plasmids and with or without TNF-α treatment. p65 (Green), mCherry (Red). n≥3 independent experiments.
(TIFF)

S2 Fig TNF-α treatment does not affect endogenous TDP-43 distribution in MCF-7 cells. In MCF-7 cells, the endogenous TDP-43 (Red) was normally located in the nucleus when p65 (Green) was trans-located into nucleus (Blue) after 30 min TNF-α treatment. n≥3 independent experiments.
(TIFF)
