# Supplementary figures and images for: TDP-43 Inhibits NF-κB Activity by Blocking p65 Nuclear Translocation
Source: PLoS One. 2015 Nov 16;10(11):e0142296. doi: 10.1371/journal.pone.0142296 (PMC4646651; doi:10.1371/journal.pone.0142296)

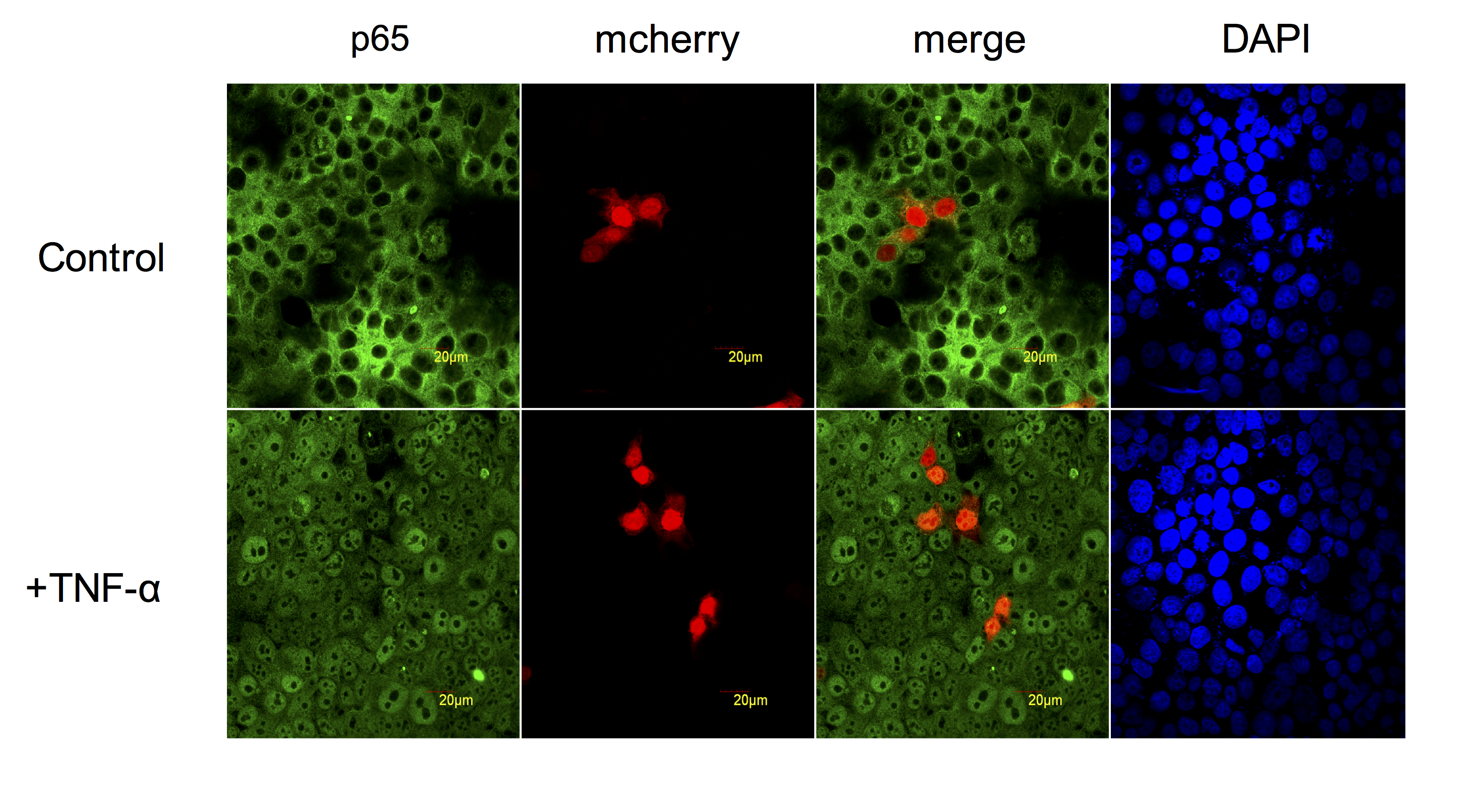

Supplement: S1 Fig — MCF-7 cells were transfected with 2μg pcDNA3-mCherry empty plasmids and with or without TNF-α treatment. p65 (Green), mCherry (Red). n≥3 independent experiments. (TIFF) [file pone.0142296.s001.tiff]

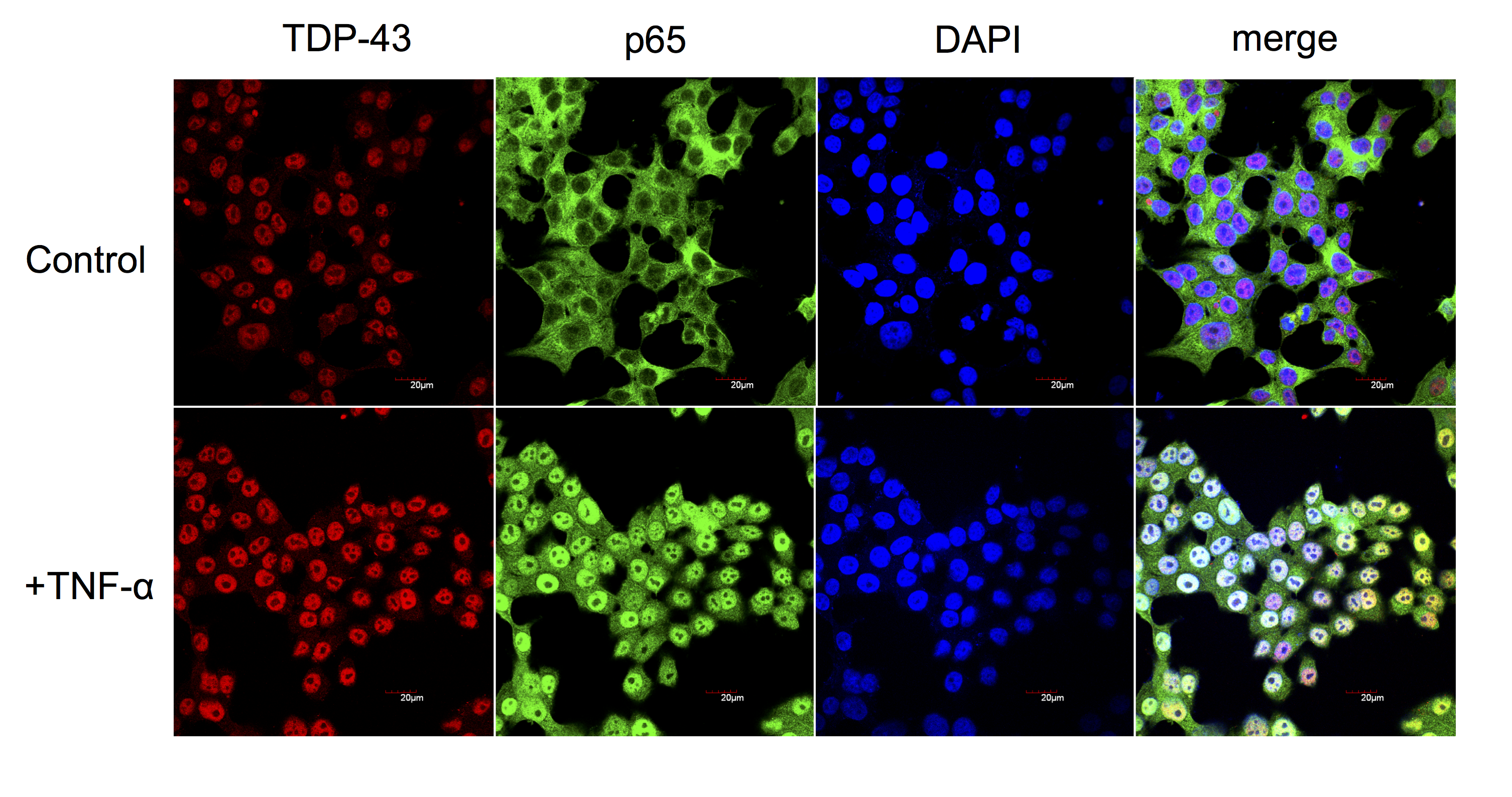

Supplement: S2 Fig — In MCF-7 cells, the endogenous TDP-43 (Red) was normally located in the nucleus when p65 (Green) was trans-located into nucleus (Blue) after 30 min TNF-α treatment. n≥3 independent experiments. (TIFF) [file pone.0142296.s002.tiff]
